# Aortic Dissection with Subsequent Hemorrhagic Tamponade Diagnosed with Point-of-care Ultrasound in a Patient Presenting with STEMI

**DOI:** 10.5811/cpcem.2019.1.40869

**Published:** 2019-02-26

**Authors:** Eric Abrams, Angela Allen, Shadi Lahham

**Affiliations:** * University of California, Irvine, Department of Emergency Medicine, Orange, California; † University of California, Irvine, School of Medicine, Irvine, California

## Abstract

A 58-year-old male with no past medical history presented to the emergency department with sudden onset left lower extremity weakness and central chest pain with radiation to his back. Electrocardiogram revealed an acute inferior and posterior ST-segment elevation myocardial infarction (STEMI). Point-of-care ultrasound (POCUS) demonstrated right ventricular akinesis consistent with infarction, and an intimal defect consistent with an aortic dissection. We determined that cardiothoracic surgery was indicated rather than left-heart catheterization and anticoagulation. Using POCUS we were able to immediately diagnose a dissection of the aortic arch and considerably alter treatment in a patient presenting with STEMI.

## INTRODUCTION

Aortic dissection (AD) is a life-threatening emergency with a mortality rate of up to 30%.^1^ Due to either a tear in the aortic intima or hemorrhage within the media, an AD occurs when blood separates the intima from the media.^2^ The dissection may extend either proximally to involve the aortic valve and coronary vessels and enter the pericardial space, or extend distally to involve the abdominal aorta.^2^ All of these situations can lead to severe decompensation and death if not addressed immediately. Presented in this case is a male with AD recognized via point-of-care-ultrasound (POCUS).

## CASE REPORT

A 58-year-old male who denied any past medical history presented to the emergency department (ED) with left lower extremity weakness for the prior 30 minutes. He stated that he had sudden onset paralysis, which prompted him to go to the ED for evaluation. On further inquiry, the patient also described having central chest pain radiating to the back that seemed to coincide with the paralysis. He denied shortness of breath or any other neurological deficits.

Upon arrival to the ED, the patient was diaphoretic, pale, and in acute distress. Vitals at the time included a pulse of 74 beats per minute, blood pressure of 166/97 millimeters of mercury in the right upper extremity, respirations of 20 breaths per minute, oxygen saturation of 97% on room air, and an oral temperature of 97.9 degrees Fahrenheit. His pertinent physical exam findings included bilateral diminished radial pulses and 0/5 strength in his left lower extremity. His lungs were clear to auscultation bilaterally and there was no appreciable murmur. No chest radiograph (CXR) was obtained.

**Image 1:**
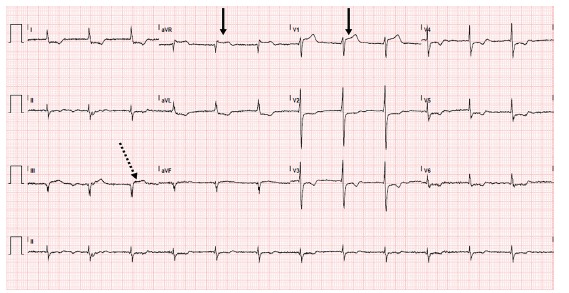
Electrocardiogram performed demonstrating inferior ST-segment elevation myocardial infarction (dashed arrow) with additional ST-segment elevation in V1 and aVR (solid arrow) consistent with right coronary artery infarction with right ventricle involvement.

**Image 2:**
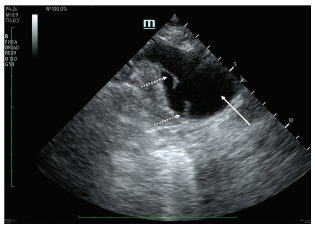
Suprasternal ultrasound visualizing the aortic arch in transection (white arrow) with a non-contiguous dissection flap at the 8 and 10 o’clock positions (dashed arrow) representing the aortic mural defect causing dissection.

An electrocardiogram (ECG) was performed (Image 1) that demonstrated an acute inferior and posterior ST-segment elevation myocardial infarction (STEMI), which prompted activation of a “code STEMI.” However, while he was still in the ED, we performed a POCUS. Ultrasound revealed right ventricle akinesis consistent with an acute MI and a dissection flap of the aortic arch, but was negative for pericardial effusion or tamponade at that time (Image 2). Based on these ultrasound findings and with cardiology at bedside, the code was cancelled as it was agreed that percutaneous coronary intervention should not be performed and instead cardiothoracic surgery be emergently paged. The patient was immediately brought to the radiology suite for an emergent computed tomography angiogram (CTA) of the chest and abdomen.

**Image 3:**
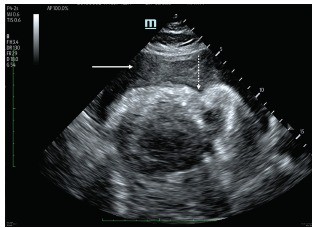
Subxiphoid cardiac ultrasound demonstrating a large complex pericardial effusion (solid arrow) with complete right ventricular (RV) collapse and bowing of the RV free wall (dashed arrow).

Unfortunately, while on the table prior to imaging acquisition, the patient became bradycardic, unresponsive, apneic, and was found to be in pulseless electrical activity arrest. CTA was aborted and cardiopulmonary resuscitation was initiated. He was brought to the resuscitation bay where repeat POCUS demonstrated a large, complex pericardial effusion with right and left ventricular collapse representing hemorrhagic tamponade (Image 3). An emergent pericardiocentesis was performed with ultrasound guidance without success. Given hemorrhagic tamponade from AD and failed pericardiocentesis, the resuscitation was deemed to be medically futile and time of death was called.

## DISCUSSION

The incidence of AD in the general population is up to six cases per 100,000 per year and up to 30 in individuals over 65.^2^ Common risk factors for this condition include longstanding arterial hypertension, connective tissue disorders, vascular inflammatory processes, deceleration trauma, and iatrogenic origins, with hypertension being the most common.^2^ There are two methods to classify AD. The Stanford classification includes type A and type B where the former involves the ascending aorta and the latter does not. The DeBakey classification is divided into three types: I involves the ascending and descending aorta; II involves only the ascending aorta; and III spares the ascending aorta and arch.^2^

**CPC-EM Capsule**

What do we already know about this clinical entity?

*Aortic dissection is a rare but life-threatening cause of ST-segment elevation myocardial infarction (STEMI)*.

What makes this presentation of disease reportable?

*The use of point-of-care ultrasound (POCUS) to cancel catheterization laboratory activation based on the diagnosis of aortic dissection*.

What is the major learning point?

*POCUS should be used in patients who present with STEMI to evaluate for other life-threatening pathology*.

How might this improve emergency medicine practice?

*If aortic dissection can be readily diagnosed with ultrasound, the new standard of care for STEMI patients may be to perform POCUS*.

The classic symptoms associated with AD vary widely, complicating diagnosis and treatment plans. In a case-control study, it was found that the absence of abrupt-onset pain could help rule out dissection while the presence of tearing or ripping pain, hypotension, pulse deficit, neurologic deficits, and new murmur can help rule in the diagnosis; however, less than 50% of patients present with classic chest pain.^3^ Pulse deficits are present in only 20% of people, and 10–15% of patients deny pain altogether.^1^–^2^ Symptoms may even resemble associated conditions such as acute MI. Although an AD may extend into the coronary vessels leading to MI, treatment of a suspected MI with anticoagulation and antiplatelet therapy is detrimental in AD and can double mortality.^4^ One study found that multiple patients with acute MI induced by AD were incorrectly treated with fibrinolysis and estimated that the mortality rate of such patients ranged from 69–100%, highlighting the importance of accurate diagnosis of the underlying cause of a patient’s condition.^5^

The American College of Emergency Physicians (ACEP) evaluated a study that attempted to generate a standard guideline for the diagnosis and treatment of these patients. They concluded that the decision and methods to evaluate for acute AD ought to be decided by individual clinicians based on their judgment since it has been reported that AD is only initially suspected in the ED 43% of the time, especially in the absence of chest and abdominal symptoms.^6^–^7^ Diagnosis can be made using several imaging modalities. ACEP recommends CTA as one of the gold standards of diagnostic imaging modalities along with echocardiogram and magnetic resonance imaging (MRI), but the latter two modalities are used sparingly due to the lack of trained physicians and limited availability of these resources.^6^

The International Registry of Acute Aortic Dissection reported that echocardiography is used 33%, CT 61%, MRI in 2%, and angiography 4% of the time. As a secondary technique, echocardiogram was used 56%, CT 18%, MRI 9%, and angiography 17% of the time. CXR is abnormal in 60–90% of suspected ADs, but acute dissections can often have normal reads. ECG is also used but can be normal or very abnormal when an ascending dissection leads to coronary compromise.^2^ Although ultrasound is not as widely used as CTA in the diagnosis of AD, it has been shown to be an effective tool in the rapid assessment of patients meeting certain criteria. In one such study, an AD POCUS protocol was developed that combined transthoracic and abdominal ultrasound. Pericardial effusion, intimal flap, and aortic outflow tract diameter data were gathered and successfully identified AD in 96.4% of patients with 100% sensitivity for Stanford type A dissections.^8^

As in this case, when a dissection extends into the coronary arteries it more commonly affects the right coronary artery and involves the inferior wall.^2^ Given the mortality rate, the use of POCUS as an immediate means to diagnose AD can be life-saving.^1^ Treatment for Stanford type A dissections is surgical, while type B patients are treated medically.^2^ Type A dissection in our patient was immediately visualized with ultrasound, which prevented the use of anticoagulation and antiplatelet therapy and altered treatment plans to surgical intervention. Although the patient succumbed to hemorrhagic tamponade, receiving dual antiplatelet therapy, anticoagulation, and undergoing an emergent left heart catheterization solely based off his ECG would have exacerbated his dire situation. Despite our patient’s outcome, the case we present adds to the evidence that POCUS is an integral component for management and treatment when evaluating a patient with chest pain in the ED.

## CONCLUSION

POCUS may be useful in the initial evaluation of patients with suspected aortic dissection. When a dissection extends into the coronary arteries leading to MI, overt presenting symptoms of MI may lead a practitioner to overlook dissection and instead treat for uncomplicated MI. This case presentation demonstrates how the use of POCUS allowed for the rapid diagnosis of aortic dissection. This diagnosis completely altered the treatment plan of the patient and prevented further interventions such as fibrinolytic therapy that would have worsened his condition.
